# A rare cutis verticis gyrata secondary to cerebriform intradermal nevus: case report and literature review

**DOI:** 10.1186/s12893-021-01229-9

**Published:** 2021-05-04

**Authors:** Weiliang Zeng, Lili Guo

**Affiliations:** 1 Department of Plastic Surgery, The First Affiliated Hospital of Zhengzhou University, 450052 Zhengzhou, China; 2 Scar Research Center, The First Affiliated Hospital of Zhengzhou University, 450052 Zhengzhou, China

**Keywords:** Cutis verticis gyrata, Cerebriform intradermal nevus

## Abstract

**Background:**

Cutis verticis gyrate (CVG) is a rare morphologic syndrome that presents with hypertrophy and folding of the scalp. CVG can be classified into three forms: primary essential, primary non-essential, and secondary. Cerebriform intradermal nevus (CIN) is a rare cause of secondary CVG. We are here to report a rare case of CVG with an underlying CIN and discuss the clinical course, treatment options, and critical screening guidelines for these patients.

**Case presentation:**

A 25 year-old male patient presented with a chief complaint of generalized hair loss in the scalp parietaloccipital region for a duration of 1 year and the hair loss area was occasionally accompanied by mild itching. The hair loss started gradually and worsened over time. In addition, he had scalp skin folds resembling the ridge and furrow of the cerebral cortex in the parietaloccipital region since birth. Physical examination revealed hypertrophy and formation of folds in the parietal-occipital area, forming 5 to 6 furrows and ridges. The size of the cerebriform mass was about 12.0 cm × 8.5 cm, without other skin lesions. Diffuse non-scarring hair loss was distributed on the posterior-parietal scalp, mid-parietal scalp and superior-occipital scalp. The diseased tissue of the patient's parietaloccipital area was excised under general anesthesia. The postoperative pathological examination of the tissue excised showed that there were dense intradermal melanocytic nevus, so the patient was diagnosed with secondary CVG caused by CIN. At the 2 year follow-up, there were no obvious changes in the lesions.

**Conclusions:**

CIN must be differentiated from other conditions that manifest as CVG, including primary essential or non-essential CVG and secondary CVG caused by other reasons. Each CIN patient requires a specific decision of whether to excise the lesion surgically or follow a wait-and-see policy, depending on the patient's will and specific condition. Surgical treatment may be performed when there is an aesthetic demand. However, clinical observation and close follow-up is also a good treatment choice for patients with stable disease or mild symptoms.

## Background

Cutis verticis gyrata(CVG) is an uncommon skin disease known for cerebriform folds and wrinkles. Alibert first proposed a clinical description in 1837, while the term CVG was proposed by Unna in 1907 [1]. Due to the rarity of this disease, the current worldwide prevalence rate is uncertain, but it is estimated that the prevalence in the male population is 1/100,000 and the prevalence in the female population is 0.026/100,000 [2]. CVG is classified into primary essential, primary non-essential and secondary forms [3]. Primary essential CVG has not known associated disorders, while primary non-essential CVG may be linked to neurological diseases, mental deficiency, or ophthalmological abnormalities. Secondary CVG is related to underlying systemic disorders, endocrine disorders, inflammatory dermatoses, neoplasms diseases, or truma, such as pachydermoperiostosis, acromegaly, myxedema, tuberous sclerosis, amyloid deposition, dermatofibroma, hamartoma, eczema, or psoriasis [4–10]. A typical histopathology of the primary CVG will reveal hypertrophy and hyperplasia of epidermal appendages and thickened dermal collagen in skin with no other histologic abnormalities [11]. In contrast, the histopathological changes of the scalp in secondary CVG shows abnormalities characteristic of the underlying etiology. Cerebriform intradermal nevus(CIN) is a rare cause of secondary CVG, accounting for 12.5% of all CVG cases [12]. This report serves as an example of secondary CVG due to a CIN, with specific focus on the diagnostic aspects and therapeutic possibilities in such cases.

## Case presentation

**Fig. 1:**
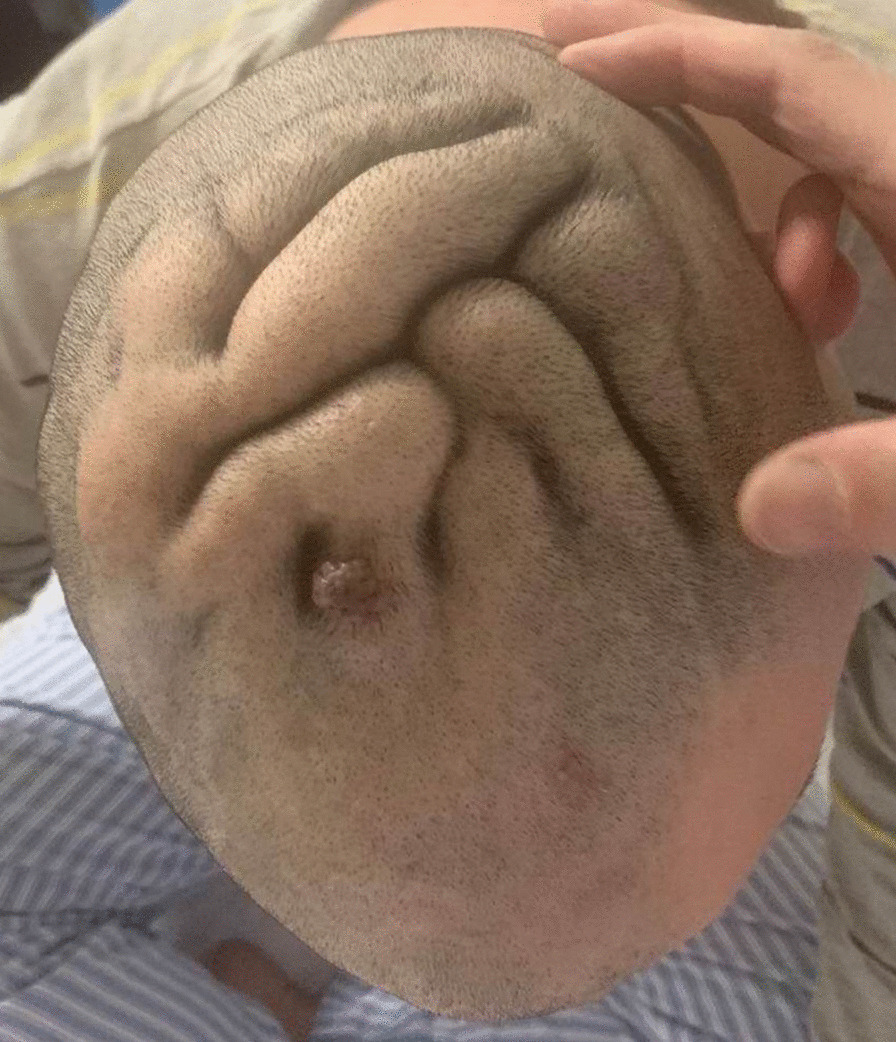
Prominent skin folds that resemble the ridge and furrow of the cerebral cortex in the parietaloccipital region

**Fig. 2:**
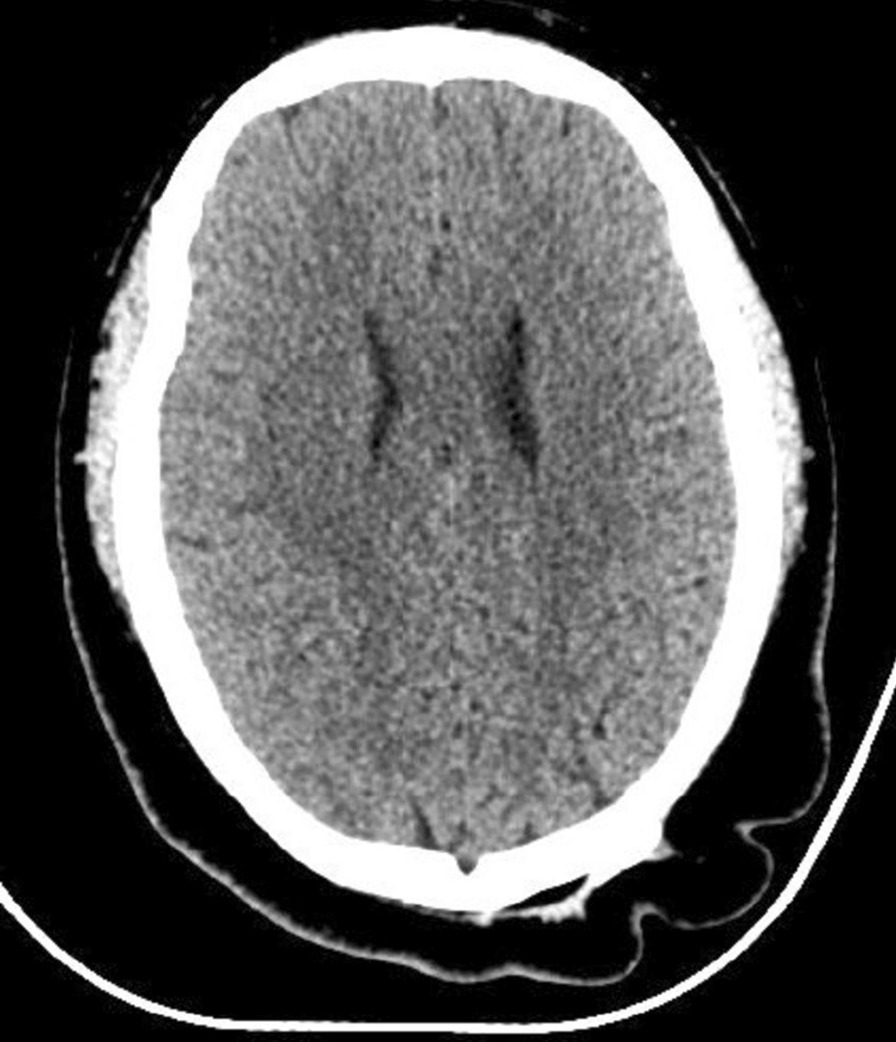
CT scan images revealed a thickened dermis and excessive growth of the scalp, forming the characteristic scalp folds, without abnormalities of bone or intracranial tissue

**Fig. 3:**
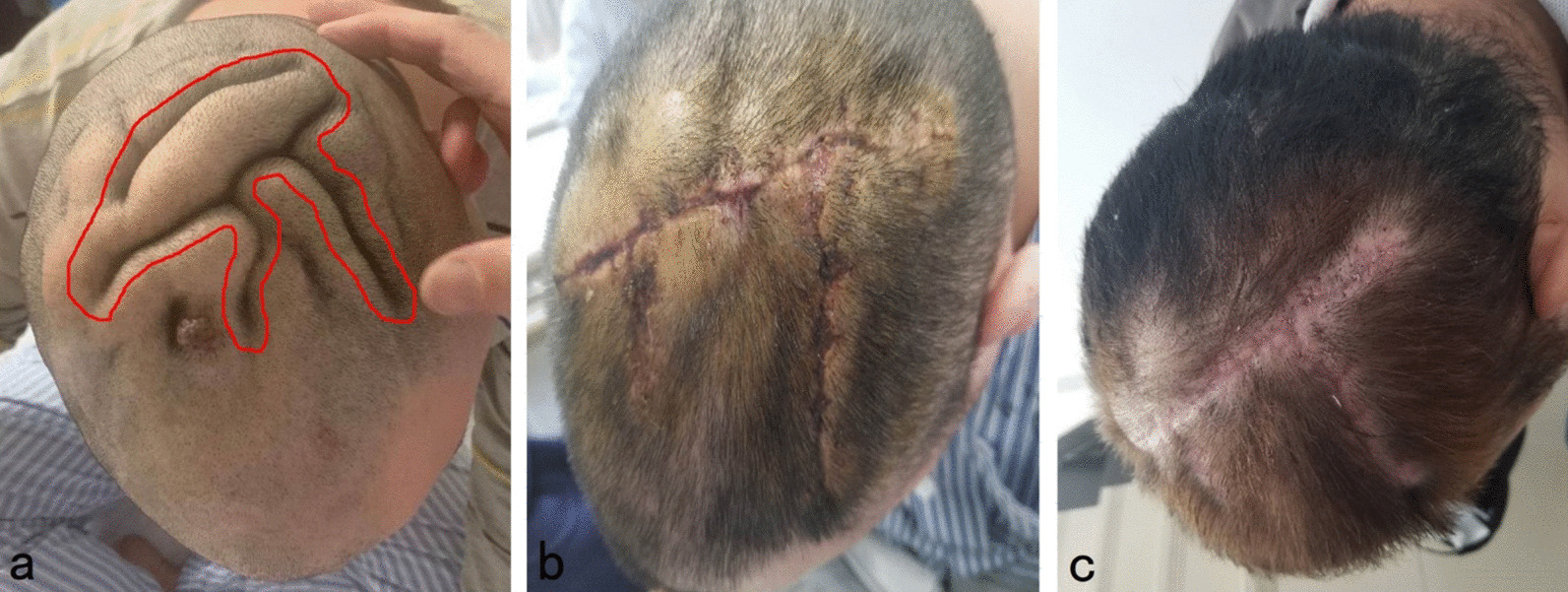
**a** A π-shaped incision was designed on the surface of the diseased tissue. **b** This was a photo taken on the 10th day after the operation. Surgical sutures had all been removed, leaving a π-shaped scar with a total length of about 18 cm. **c** A photo taken 1 month after the operation

**Fig. 4:**
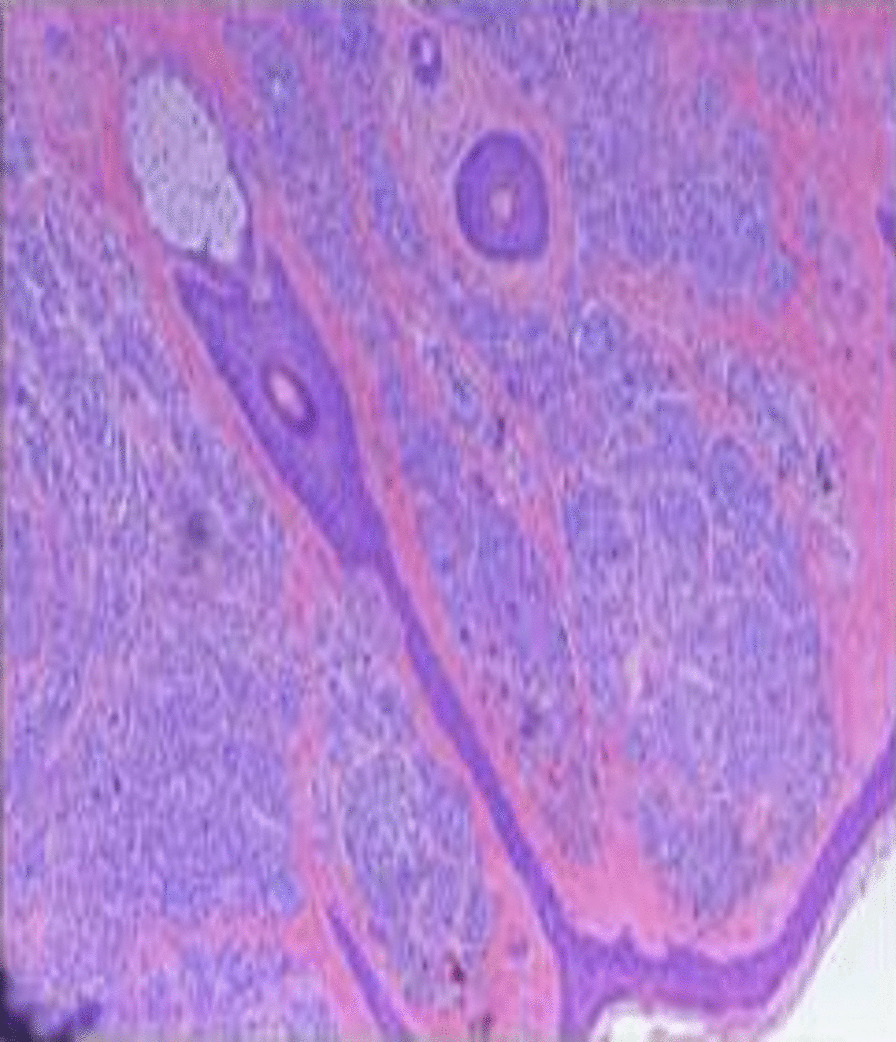
There were background densely arranged melanocytic nevus cells in the dermis

A 25 year-old male patient presented with a chief complaint of generalized hair loss in the scalp parietaloccipital region for a duration of 1 year, saying that hair loss started gradually and had worsened over time. In addition, this patient indicated that the alopecia area was occasionally accompanied by mild itching. He had scalp skin folds resembling the ridge and furrow of the cerebral cortex in the parietaloccipital region since birth. These were a small protuberant mass at first and had gradually increased in size and volume in proportion to his growth. He was of normal intelligence and denies neuropsychiatric diseases, eye diseases, scalp injuries, endocrine diseases, tumors, chronic dermatitis and similar family history. Physical examination at our department revealed hypertrophy and formation of folds in the parietal-occipital area, forming 5 to 6 furrows and ridges (Fig. 1). The size of the cerebriform mass was about 12.0  × 8.5 cm, without other skin lesions. Diffuse non-scarring hair loss was distributed on the posterior-parietal scalp, mid-parietal scalp and superior-occipital scalp. Further examination did not reveal any changes in neurology or ophthalmology, swelling of the maxillofacial or limb joints, skin thickening and tissue growth. A plain head computed tomography (CT) scan revealed a thickened dermis and excessive growth of the scalp, forming the characteristic scalp folds, with no associated involvement of the bone or intracranial tissue (Fig. 2). Due to the lack of neurological or psychiatric findings, this patient was not referred for brain magnetic resonance imaging (MRI). All laboratory tests (routine blood count, glycemia, lipid, basic metabolic profile, liver functions, kidney functions, immune function, insulin-like growth factor 1, growth hormone, parathyroid hormone, cortisol and acromegaly screening) were within normal range. The patient did not accept our suggestion about performing a scalp skin biopsy firstly. In contrast, due to the strong desire to improve the appearance, he and his families chose surgical excision diretly. After the hospitalization procedures, completing the relevant examination and eliminating the contraindications of the operation, the diseased tissue of the patient's parietaloccipital area was excised under general anesthesia. During the operation, a π-shaped incision was designed on the surface of the diseased tissue (Fig. 3a). The skin, subcutaneous tissue, subcutaneous superficial fascia layer and cap aponeurosis layer were cut off successively along the designed incision line with a blade. The diseased tissue was peeled off with an electric knife in the cap aponeurosis layer. Then the subcutaneous tissue on both sides of the incision was separated to form two fascia tissue flaps. After using an electric knife to fully stop the bleeding and indwelling a negative pressure drainage tube, we pulled the bilateral fascia tissue flaps to the middle to suture the incision interruptly with 4-0 absorbable thread and performed an interrupted suture of the skin with 5-0 silk thread. After the operation, the incision healed well, leaving only a π-shaped scar with a total length of about 18 cm (Fig. 3b, c). Pathological examination of the tissue excised during the operation showed that there were dense intradermal melanocytic nevus (Fig. 4). In light of these findings, the patient was diagnosed with secondary CVG caused by CIN. At the 2 year follow-up, there were no obvious changes in the lesions.

## Discussion and conclusions

**Fig. 5:**
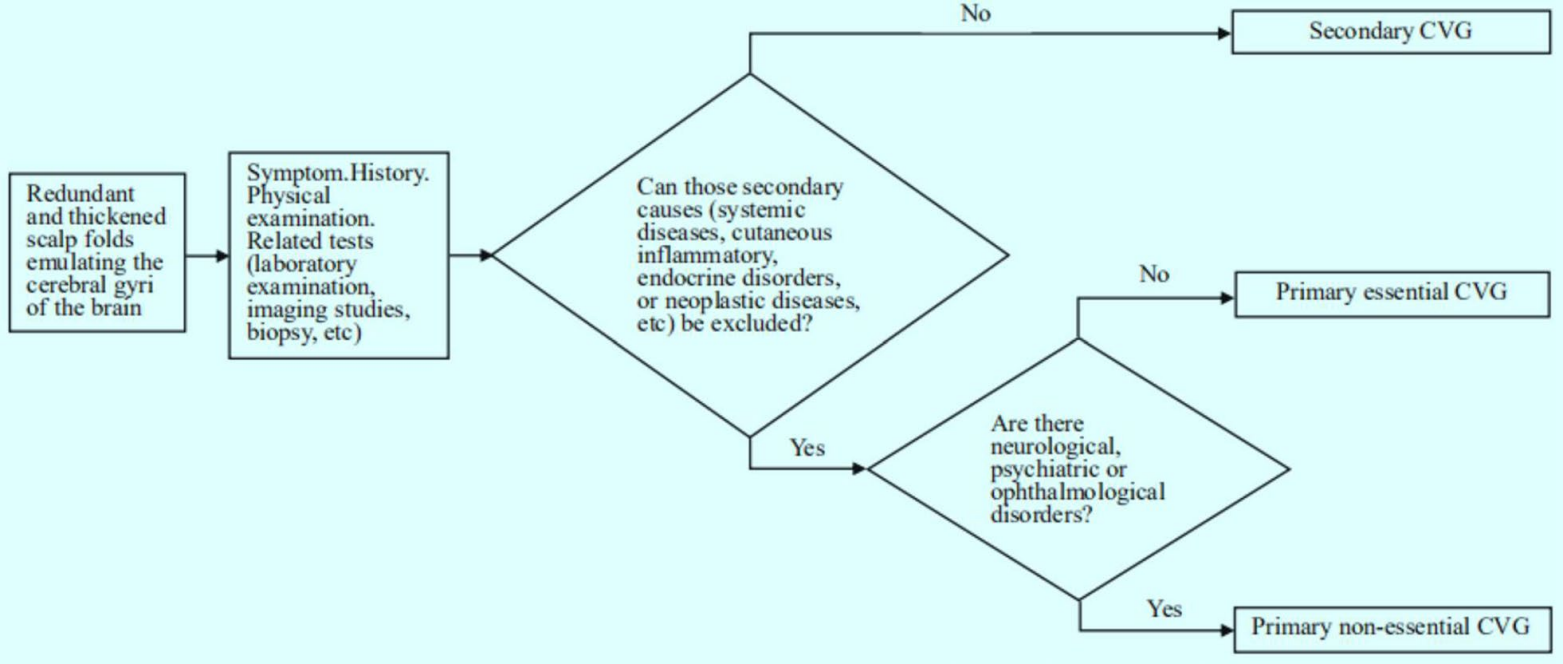
Diagnostic algorithm

CVG is a disease characterized by the formation of sulci and gyri that resemble those of the cerebral cortex. CVG is categorized as primary essential, primary non-essential, or secondary depending on the intrinsic etiology. Primary essential CVG is an isolated discovery and has nothing to do with any neurological or ophthalmological disease. This type usually predominates in male patients during or after puberty and is usually distributed symmetrically, mainly affecting the vertex and occipital region [13]. Primary non-essential CVG is usually associated with neurological and ophthalmological disorder, such as intellectual disability, epilepsy, deafness, cataracts, strabismus and blindness [14]. The clinical presentation of secondary CVG varies depending on the underlying cause, including hormone-related diseases such as acromegaly, pituitary tumors, thyroid diseases, or myxedema; cutaneous inflammatory such as tuberous sclerosis, eczema, psoriasis, acne conglobate, or Darier disease; infectious diseases such as syphilis or human immunodeficiency virus; neoplasms diseases such as leukemia, or T-cell lymphoma [4, 5, 7, 10, 15, 16]. The histopathology of the primary CVG will reveal hypertrophy and hyperplasia of epidermal appendages and thickened dermal collagen in skin, whereas the histopathological changes in secondary CVG shows abnormalities characteristic of the underlying etiology [11]. The pathogenesis of primary CVG are still unclear. Although some familial forms with autosomal dominant condition or recessive inheritance with variable expression have been reported, the majority of cases of primary essential CVG are sporadic [17]. Due to the hyperplasia of dermal collagen in CVG, some authors believe that it is an autosomal dominant condition caused by mutation in the FGFR [18]. The FGFR2 gene encodes a transmembrane tyrosine kinase and can function as a mitogenic, angiogenic or inflammatory factor, which may be involved in the pathological process of CVG [19]. It is not difficult to make a diagnosis of CVG based on clinical manifestations, but it is necessary to draw our attention to distinguish these three forms. For any of the above three forms of CVG, the other two must be excluded before making the final diagnosis (Fig. 5). In most cases, primary CVG is asymptomatic, except for some neurological and ophthalmological symptoms. In addition, the operation is invasive, so clinical observation is a good treatment choice for patients with stable disease or mild symptoms. However, surgical treatment, such as partial excision, staged excision, skin grafting, local flap grafting, free flap grafting and tissue expansion may also be performed when there is an aesthetic demand. For small and local lesions, partial scalp excision and skin or flap grafting can be selected. Tissue expansion and staged excision are excellent options for larger scalp lesions. Some scholars once reported a technique combining a “butterfly”-shaped scalp skin excision design and sub-galeal scalp relaxation incisions to effectively improve the appearance of the scalp [20]. In contrast, secondary CVG usually regresses after treatment of the underlying disease, although surgical excision may be necessary sometimes.

**Fig. 6:**
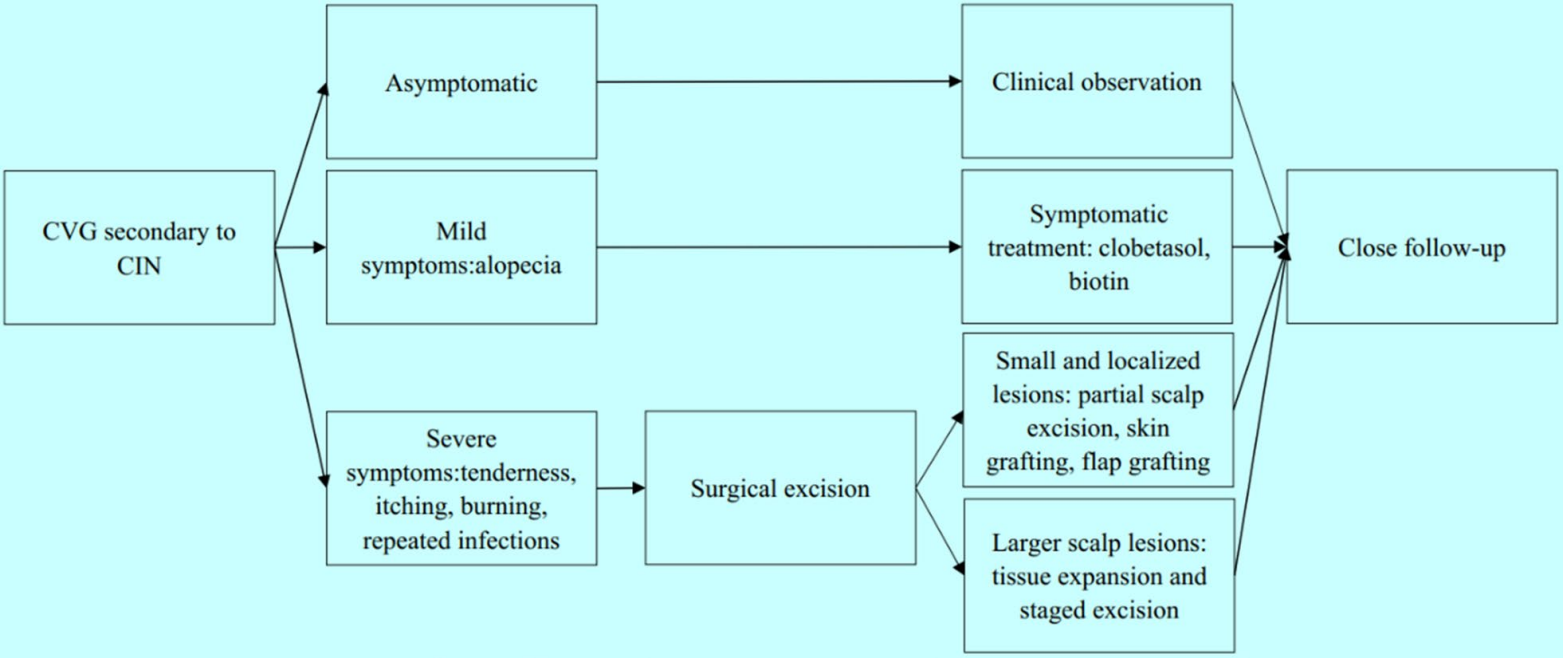
Management paradigm

The postoperative pathological results of the above patient we reported showed that there were dense intradermal melanocytic nevus, therefore, the patient was diagnosed with secondary CVG caused by CIN. CIN is one of several benign tumors that cause CVG, accounting for 12.5% of CVG cases and there is a female predilection [12]. Hammond first described a cerebriform naevus resembling CVG in 1937 [21]. It is usually congenital but can also be acquired. CIN most commonly occurs in the parietal and occipital areas of the scalp, and the frontal and temporal areas are less involved [22]. These nevus appear as a small spot, a small protuberant mass, or a patch of alopecia at birth or early in life. Over time, the lesions slowly expand and become more prominent, which was consistent with our patient's condition. The size of CIN lesions usually ranges from 3.0 × 2.0 cm to 25.0 × 22.5 cm [23]. Patients often present with progressive alopecia, occasionally accompanied by tenderness, itching, burning, and repeated infections. In the case we reported, the hair loss area of the patient was only accompanied by mild itching, and there was no tenderness, burning, or ulcer infection. The diagnosis of CIN is based on clinical and histopathological examinations. Therefore, CIN must be differentiated from other conditions that manifest as CVG, including primary CVG and secondary CVG caused by other reasons. CIN may evolve into malignant melanoma(MM), which can even appear early in life. Orkin reviewed 50 cases of CIN reported up to 1974 and found that two cases of MM were derived from CIN. One of them was a 6-year-old boy whose entire lesion was excised and grafted, and there was no sign of recurrence 11 years later [12]. The other was a 51-year-old woman who died 10 months later, despite chemotherapy and radiotherapy. Hayash reviewed 17 cases of CIN reported from 1974 to 2008 with one case of MM from CIN. This patient was a 66-year-old man whose entire lesion was excised and received chemotherapy, with no recurrence during a 6-month follow-up. Therefore, Hayashi found the frequency of MM alteration of CIN to be 4.5% (3 of 67 cases) [24]. Here we searched the relevant available literature reported from 2008 to 2020 from three databases: PubMed, Web of Science, and the Cochrane Library. Search terms used were 'cutis verticis gyrata' or 'cerebriform intradermal nevus'. The language was restricted to English. Furthermore, we reviewed reference lists from retrieved articles to appraise other potential studies. In finally, we found a total of ten CIN patients, with no case of MM from CIN (Table 1). Including these added cases, we estimated that the risk of CIN transforming to MM was 3.9% (3 of 77 cases). Even though, we must realize that there is no a precise and completed computerized search for all relevant available articles published up to 2020. The calculation of malignant transformation risk is not so accurate that more future cohort studies or a true systematic review are needed. Because of the the possibility of developing MM and aesthetic demand, surgical excision of the lesion is recommended. However, considering the low risk of malignant transformation of CIN and the surgical excision of the lesion usually involves extensive mutilation, Van Geest et al. recommended a wait-and-see policy [35]. Nevertheless, for those CIN patients who have not undergone surgery to excise the lesion, close follow-up is still very necessary (Fig. 6).

**Table 1:** Summary of clinical characteristics of previously reported cases of cutis verticis gyrata secondary to cerebriform intradermal nevus

Author	Year	Age	Sex	Lesion regions	Lesion size	Accompanying symptoms	Treatment options	Follow-up
Fronek LF et al.[25]	2019	46	Female	Posterior parietal, Middle parietal, Right superior occipital	N/A	Hair loss	Clinical observation	No recurrence
Mutlu OO et al.[26]	2016	18	Male	Posterior parietal, Middle parietal, Occipital	30 × 29 cm	No	Free flap grafting	No recurrence
Sarkar S et al.[27]	2014	28	Male	Occipital	20 × 15 cm	Hair loss, Blue nevus, Hyperpigmention nodules	Serial excision, Free flap grafting	No recurrence
Huerta M et al.[28]	2014	45	Male	Right parietal, Right temporal, Right occipital	30 × 29 cm	No	Clinical observation	No recurrence
Ghosh SK et al.[29]	2012	22	Male	Occipital	20 × 12 cm	Hair loss	Clinical observation	No recurrence
Tagore KR et al.[30]	2011	14	Male	Occipital, Left parietal	20 × 12 cm	Hair loss	Full thickness skin grafting	No recurrence
Zhang M et al.[31]	2011	17	Female	Occipital	30 × 22 cm	Hair loss	Local flap grafting	No recurrence
Muhlhoff C et al.[32]	2010	38	Female	Right parietal, Right frontal	13 × 7 cm	Hair loss, Blue nevus	N/A	No recurrence
Bonalumi FA et al.[33]	2010	43	Female	Right temporal, Right occipital	N/A	Hair loss, Seborrheic dermatitis, Fetid odor	N/A	No recurrence
Alcántara GJ et al.[34]	2010	48	Male	Left parietal, Temporal	N/A	Hair loss, Hyperpigmention nodules	Clinical observation	No recurrence

*N/A* not available

## Data Availability

The datasets used and/or analysed during the current study available from the corresponding author on reasonable request.

## Abbreviations

CVG: Cutis verticis gyrata
CIN: Cerebriform intradermal nevus
CT: Computed tomography
MRI: Magnetic resonance imaging
MM: Malignant melanoma
